# The London Panel Committee

**Published:** 1914-11-14

**Authors:** 


### The London Panel Committee.

### TUESDAY'S DECISIONS.

A special meeting of the London Panel Committee was held on November 10.

### Panel Practitioners and Tuberculosis.

The Panel Service Sub-committee reported that a deputation had waited upon the Local Government Board to discuss the relations of practitioners on the panel with tuberculosis consulting officers. The deputation urged that all insured persons suffering from tuberculosis and not resident in institutions should be under the general care of their panel practitioners, and that the services provided for them at a dispensary should be mainly consultative and given in co-operation with the practitioner on the panel. The point was emphasised that if the panel practitioner was required to submit periodical reports to the dispensary officer the latter should also supply the practitioner with reports.

Dr. E. Hudson, chairman of the Sub-committee, mentioned that the representatives of the Local Government Board agreed that the position of the dispensary officer should be consultative, and that an insured person should not be received at a dispensary without the knowledge of the practitioner responsible for his treatment.

Dr. Bernstein urged the great importance of retaining tuberculosis treatment, as far as possible, in the hands of the general practitioner. In his opinion, although no fees might be obtainable, general practitioners ought to examine "contacts." Dr. Cunnington expressed agreement with this view.

### Breaches of Medical Etiquette.

The Panel Service Sub-committee also stated that in view of irregularities in the transfer of insured persons which took place at the end of the medical year 1913 the Panel Committee should report to the proper authority any case in which a practitioner exhibited publicly notices regarding the date of revision, printed or circulated transfer forms, canvassed in any manner whatsoever for patients, or secured the transfer to his list of large numbers of insured persons on the lists of practitioners absent on military duty. The Committee passed a resolution endorsing this view.

### The Position of Medical Partnerships.

A legal opinion, given by Mr. Gore Browne, K.C., was reported to the Committee upon the subject of the legal position of practitioners who are members of a firm or partnership of practitioners. Mr. Gore Browne expressed the view that under the Insurance Act an agreement for the treatment of insured persons purporting to deal with a firm as such would be irregular. The regulations were inconsistent with agreements being made with firms as such, and by inference prohibited them.

### Cases of Excessive Prescribing.

The Committee approved a number of decisions given by the Pharmacy Sub-committee in cases in which the Pharmaceutical Committee alleged excessive prescribing. Five cases were not dealt with, as the practitioners were absent on military duty. In seven cases it was held that the period under review was not sufficiently extensive for a decision to be made. In seventeen cases it was found that the cost of the drugs and appliances ordered was in excess of reasonable requirements, and in nine cases the cost was held to be not in excess.

Dr. C. W. Hogarth, the chairman of the Sub-committee, stated that in many cases the doctor was able to give a satisfactory explanation. Some practitioners, called up in reference to the ordering of proprietary articles, had been able to produce letters from the Insurance Committee stating that the preparations might be used. In other cases practitioners had been misled by travellers from wholesale houses who assured them that certain proprietary articles could be used. A further 200 cases had been remitted by the Pharmaceutical Committee.

Dr. Bernstein desired to discuss the methods followed by the Sub-committee in pursuing its inquiry, but the Chairman ruled this out of order.

### Reduction of Lists Due to the War.

The Panel Service Sub-committee noted that no figures were available as to the number of insured persons in London serving with the Forces. If the number equalled one-sixth of the insured population 250,000 persons were no longer entitled to medical and sanatorium benefits. Unless the necessary adjustments were made before December 31, 1914, practitioners would be credited in respect of persons on their lists, whether serving with the Colours or not. No doubt a number of practitioners would suffer considerably by the reduction of their lists, but the rights of individual practitioners would not permit an arrangement whereby the depleted medical benefit fund should be distributed on the basis of the numbers on lists before the war. The Insurance Committee should therefore make the necessary alterations in the lists forthwith.

